# Supplementary material for: First report of leopard fossils from a limestone cave in Kenting area, southern Taiwan
Source: PeerJ. 2021 Aug 23;9:e12020. doi: 10.7717/peerj.12020 (PMC8388558; doi:10.7717/peerj.12020)
Supplement: Supplemental Information 2 [file peerj-09-12020-s002.docx]

**Supplemental Table 2** Quantities of the measurements.

|  | linear measurements | | | geomorphometric measurement | | |
| --- | --- | --- | --- | --- | --- | --- |
|  | p3 | p4 | m1 | p3 | p4 | m1 |
| Kenting fossils | 1 | 1 | 1 | 1 | 1 | 1 |
| Modern clouded leopards  (*Neofelis* sp.) | 13 | 12 | 12 | 15 | 12 | 16 |
| Modern tigers  (*Panthera tigris*) | 9 | 10 | 9 | 25 | 25 | 28 |
| Modern leopards  (*Panthera pardus*) | 5 | 4 | 7 | 9 | 8 | 11 |
