# Supplementary material for: First report of leopard fossils from a limestone cave in Kenting area, southern Taiwan
Source: PeerJ. 2021 Aug 23;9:e12020. doi: 10.7717/peerj.12020 (PMC8388558; doi:10.7717/peerj.12020)
Supplement: Supplemental Information 3 [file peerj-09-12020-s003.docx]

**Supplemental Table 3** Factor loadings for selected ratios of p3, p4, and m1

|  | p3 | | |  | p4 | | | | | |  | m1 | | | | | | |
| --- | --- | --- | --- | --- | --- | --- | --- | --- | --- | --- | --- | --- | --- | --- | --- | --- | --- | --- |
|  | A1 | B1 | C1 |  | A2 | B2 | C2 | D2 | E2 | F2 |  | A3 | B3 | C3 | D3 | E3 | F3 | G3 |
| PC1 | -0.153 | -0.345 | -0.925 |  | -0.540 | -0.264 | 0.578 | 0.517 | -0.016 | 0.187 |  | 0.222 | -0.253 | -0.165 | 0.019 | 0.795 | -0.459 | -0.118 |
| PC2 | -0.723 | -0.677 | -0.132 |  | 0.016 | -0.784 | -0.073 | -0.230 | 0.552 | -0.145 |  | 0.213 | 0.326 | -0.022 | -0.115 | 0.492 | 0.767 | 0.043 |
| PC3 | 0.673 | -0.649 | -0.353 |  | 0.093 | -0.005 | 0.641 | -0.418 | -0.254 | -0.583 |  | 0.050 | -0.535 | 0.102 | -0.633 | -0.098 | 0.212 | -0.439 |
